# Research on the Material Removal in the Polishing of Potassium Dihydrogen Phosphate Crystals Based on Deliquescent Action

**DOI:** 10.1155/2014/949012

**Published:** 2014-01-02

**Authors:** Shaolong Guo, Feihu Zhang, Yong Zhang, Dianrong Luan

**Affiliations:** ^1^State Key Laboratory of Tribology, Tsinghua University, Beijing 100084, China; ^2^School of Mechatronics Engineering, Harbin Institute of Technology, Harbin 150001, China

## Abstract

Through the polishing experiments of potassium dihydrogen phosphate (KDP) crystals based on deliquescent action, the effect of several major factors, including crystal's initial surface state, polishing time, and revolution of polishing plate, on material removal was researched. Under certain experimental conditions, the rules of material removal were reached, and experimental results are discussed, which lays the foundation for popularization and application of polishing technology for KDP crystals based on deliquescent action.

## 1. Introduction 

In recent years, with applying high power laser systems in important technologies, including controlled thermonuclear reactions and simulating nuclear explosions, as preferred material of frequency converters and electrooptic switches in Inertial Confinement Fusion (ICF) [1], potassium dihydrogen phosphate (KDP) crystal has attracted scientists' widespread attention. KDP crystal exhibits not only a biggish photoelectric coefficient, a biggish nonlinear optical coefficient, and a high laser-induced damage threshold but also high optical uniformity and a low optical absorption coefficient [2]. However, KDP crystal is fragile, soft, deliquescent, anisotropic, and sensitive to temperature change, which makes it one of the crystalline materials most difficult to fabricate [3–5].

In many countries, scientific researches on precision and ultraprecision machining have been done in order to meet the requirements of high precision optical elements made of KDP crystals in some fields. At present, single point diamond turning (SPDT) technology is a feasible method usually used for ultraprecision machining KDP crystals [6, 7]. Fuchs et al. [8] got a surface roughness of better than 0.8 nm rms on a test sample by using single point diamond turning technology with certain machine and tool parameters. But this technology has a defect, that is, small periodic ripples produced in machining KDP crystals [9]. To overcome the defect, we proposed a new technology, that is, polishing technology for KDP crystals based on deliquescent action, which utilizes deliquescent action for ultraprecision machining KDP crystals. The polishing process of this technology can be well controlled, and the surface roughness of the KDP crystal polished with this technology reaches nanometer level. In this paper, through the polishing experiments of KDP crystals based on deliquescent action, the effect of several major factors on material removal was researched, and the experimental results are discussed.

## 2. Polishing Technology for KDP Crystals Based on Deliquescent Action

**Figure 1:**
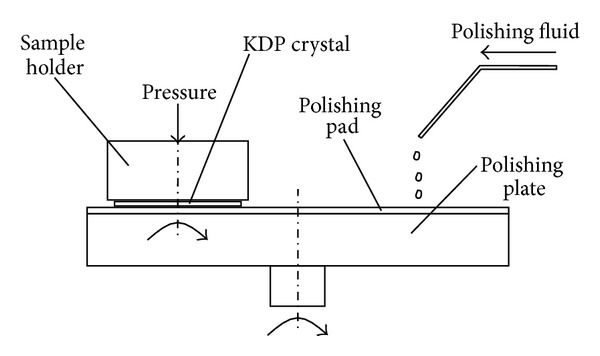
Operating principle of polishing technology for KDP crystals based on deliquescent action [11].

Generally speaking, existing polishing technologies are based on abrasives, and the abrasives will produce scratches on the surface of workpiece. Based on the fact and according to the characteristic that KDP crystal is deliquescent [10], we proposed a new ultraprecision machining technology for KDP crystals, that is, polishing technology for KDP crystals based on deliquescent action, which utilizes deliquescent action for ultraprecision machining KDP crystals. The operation principle of polishing technology for KDP crystals based on deliquescent action is shown in Figure 1 [11].

In polishing of KDP crystals based on deliquescent action, along with the polishing plate the polishing pad revolves with the same angular velocity, along with the sample holder the KDP crystal revolves with the same angular velocity, polishing fluid with water is dropped onto the polishing pad, and the revolutions of the polishing plate and the sample holder spread the polishing fluid over the polishing pad; at the same time, on the one hand the polishing fluid with water is used to exert deliquescent action on KDP crystals' surfaces, and on the other hand the polishing pad is used to exert mechanical action on KDP crystals' surfaces, and the mechanical action of the polishing pad removes the deliquescent layers of KDP crystals' surfaces; thus the ultraprecision machining of KDP crystals is achieved [11].

## 3. Experimental Material and Method

**Figure 2:**
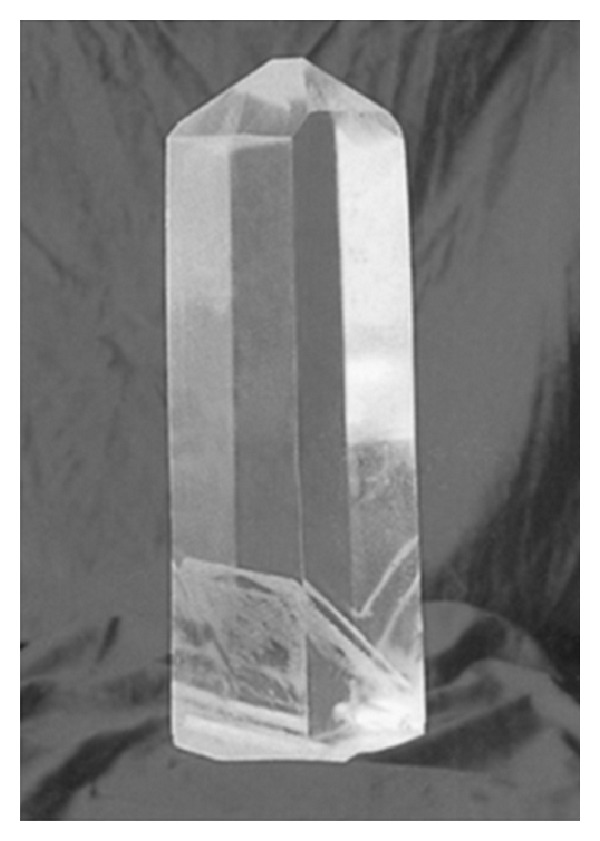
Photograph of a KDP crystal [12].

KDP crystal is a kind of good electrooptic nonlinear optical material developed in the 1940s. It belongs to tetragonal system at room temperature. The ideal shape of KDP crystal is a combination of a tetragonal prism and a tetragonal bipyramid.  Figure 2 [12] shows the photograph of a KDP crystal.

In this paper, every experiment was carried out on the type I matching surface of KDP crystal. Before the polishing of KDP crystals based on deliquescent action, the surfaces of KDP crystals were preprocessed by SPDT.

In the polishing of KDP crystal based on deliquescent action, the computation of material removal rate is an important problem. In the experiments, the thicknesses of KDP crystals before and after polishing were measured with a thickness detector; then the thicknesses were processed to obtain the material removal rates.

In this paper, to research the effect of every major factor on material removal, single-factor experiments were carried out.

## 4. Results and Discussions

### 4.1. Effect of KDP Crystal's Initial Surface State on Material Removal Rate

**Figure 3:**
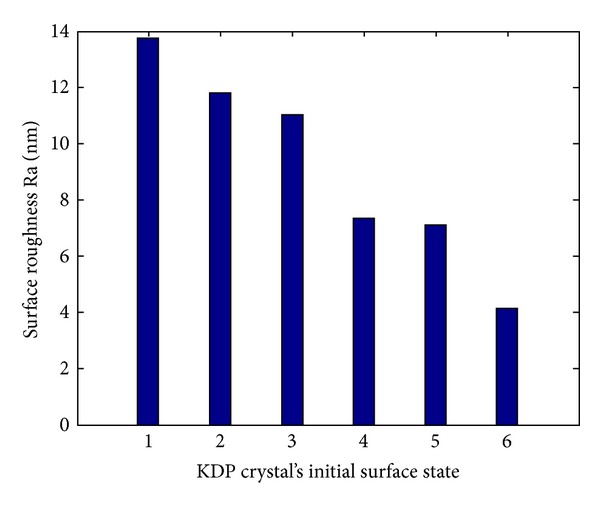
Surface roughnesses of the crystals before polishing of KDP crystals based on deliquescent action.

KDP crystal's initial surface state is represented by the surface roughness of the crystal before polishing, and an AFM is used for measuring the surface roughness. Before polishing of KDP crystals based on deliquescent action, surface roughnesses of the crystals are shown in Figure 3. In Figure 3, the numbers along the *x*-axis are initial surface state numbers.

**Figure 4:**
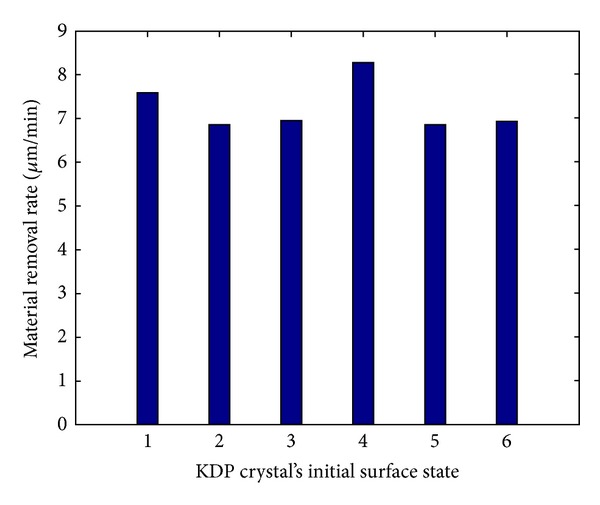
Relation between material removal rate and initial surface state of KDP crystal.

Several processing parameters in the experiments were as follows: revolution of drip *n*_*d*_ = 10 rpm, revolution of polishing plate *n*_*p*_ = 35 rpm, polishing time *t* = 10 min, and polishing pressure *p* = 0.1302 MPa. Experimental results are shown in Figure 4.

It can be seen from Figures 3 and 4 that, with reducing the surface roughness before polishing, the material removal rate does not simply rise or decrease but sometimes decreases and sometimes rises. So KDP crystal's initial surface state has no effect upon material removal rate. The reason for this is that, in the polishing of KDP crystals based on deliquescent action, the deliquescent action of the polishing fluid with water is so strong that the differences among KDP crystals' initial surface states are submerged rapidly.

### 4.2. Effect of Polishing Time on Material Removal Rate

**Figure 5:**
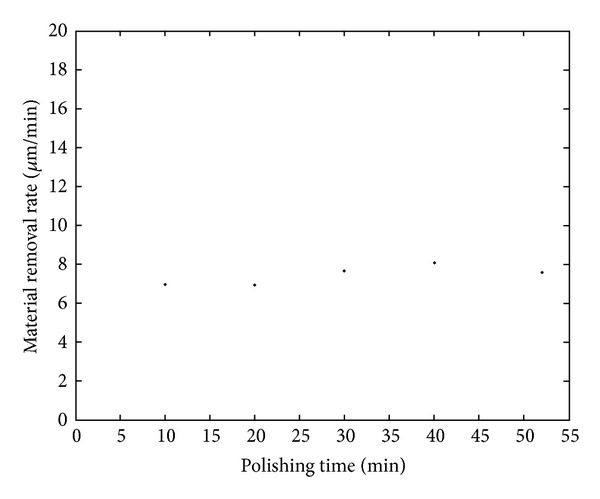
Relation between material removal rate and polishing time.

In order to research the effect of polishing time on material removal rate, the polishing experiments of KDP crystals based on deliquescent action were performed. Several processing parameters in the experiments were as follows: revolution of drip *n*_*d*_ = 10 rpm, revolution of polishing plate *n*_*p*_ = 35 rpm, polishing pressure *p* = 0.1302 MPa, and polishing time *t* = 10 min, 20 min, 30 min, 40 min, and 52 min. Experimental results are shown in Figure 5.

It can be seen from Figure 5 that, when polishing time *t* is 10 min, 20 min, 30 min, 40 min, and 52 min, respectively, the material removal rate changes in the range of 6.95 *μ*m/min~8.08 *μ*m/min, and the changes are small. This shows that the material removal rate in the polishing of KDP crystals based on deliquescent action has good repeatability and high stability.

### 4.3. Effect of Polishing Time on Material Removal Volume

**Figure 6:**
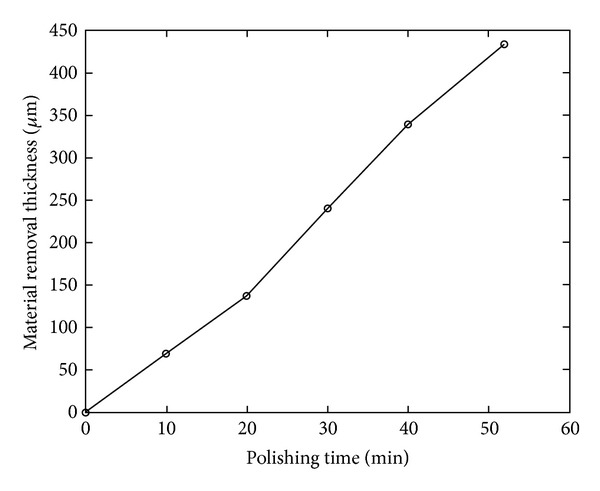
Relation between material removal thickness and polishing time.

To research the effect of polishing time on material removal volume, the polishing experiments of KDP crystals based on deliquescent action were performed. In this paper, the material removal volume is represented by the material removal thickness. Several processing parameters in the experiments were as follows: revolution of polishing plate *n*_*p*_ = 35 rpm, revolution of drip *n*_*d*_ = 10 rpm, polishing pressure *p* = 0.1302 MPa, and polishing time *t* = 10 min, 20 min, 30 min, 40 min, and 52 min. Experimental results are shown in Figure 6.

It can be seen from Figure 6 that material removal thickness is approximately linear with polishing time; that is, material removal volume is approximately linear with polishing time, and the material removal volume rises with the raising of the polishing time. So, in the polishing of KDP crystal based on deliquescent action, basically the quantitative removal of KDP crystal material can be achieved through controlling polishing time.

### 4.4. Effect of Revolution of Polishing Plate on Material Removal Rate

**Figure 7:**
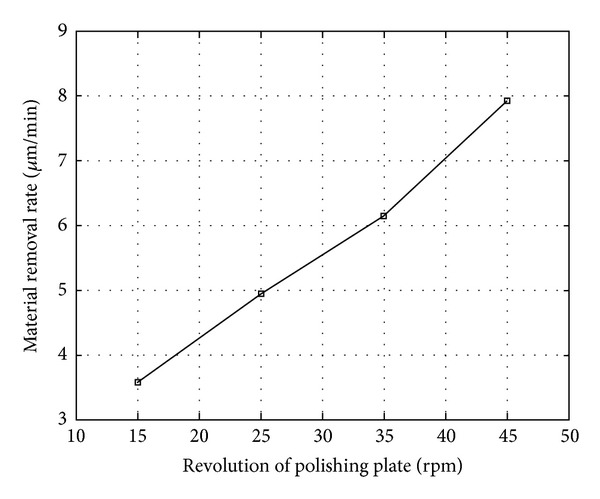
Relation between material removal rate and revolution of polishing plate.

Several processing parameters in the experiments were as follows: revolution of drip *n*_*d*_ = 10 rpm, polishing pressure *p* = 0.1302 MPa, polishing time *t* = 20 min, and revolution of polishing plate *n*_*p*_ = 15 rpm, 25 rpm, 35 rpm, and 45 rpm. Experimental results are shown in Figure 7.

It can be seen from Figure 7 that the material removal rate rises with the raising of the revolution of the polishing plate. The reason for this is that, when the revolution of the polishing plate is raised, the mechanical action of the polishing pad is getting stronger.

## 5. Conclusions

In this paper, through the polishing experiments of KDP crystals based on deliquescent action, the relations between material removal and several major factors were researched; some conclusions on the existing conditions are drawn as follows.

1. With the same polishing time, the material removal rate does not simply rise or decrease with the reducing of the surface roughness before polishing but sometimes decreases and sometimes rises. So KDP crystal's initial surface state has no effect upon material removal rate.
2. When polishing time *t* is 10 min, 20 min, 30 min, 40 min, and 52 min, respectively, the material removal rate changes in the range of 6.95 *μ*m/min~8.08 *μ*m/min; the changes are small. This shows that the material removal rate in the polishing of KDP crystals based on deliquescent action has good repeatability and high stability.
3. Material removal volume is approximately linear with polishing time, and the material removal volume rises with the raising of the polishing time. Basically the quantitative removal of KDP crystal material can be achieved through controlling polishing time.
4. The material removal rate rises with the raising of the revolution of the polishing plate.
